# Chloral Hydrate

**Published:** 1881-03

**Authors:** H. H. Kane

**Affiliations:** 191 West Tenth St., New York City; New York


### Original Communications.

ARTICLE IV.

CHLORAL HYDRATE. By H. H. KANE, M.D., New York.

*Chloral Hydrate in the Treatment of Tetanus.*—At the time when chloral hydrate was first introduced to the profession, and when it was being set forth as a cure-all for every known disease, it was tried in tetanus, and succeeded in effecting a cure in five cases in the hands of Verneuil,* and in other cases in the hands of other men. There were those then who, not content to give the drug a fair trial, prematurely proclaimed it as a specific for this affection. There are those at the present day who look upon it in the same light. That chloral is one of the most efficient drugs ever used in tetanus there can be no question, but that it is the remedy of remedies, and to be used to the exclusion of all other treatment, is absurd. Like other drugs that have been used in this affection, it succeeds best in those cases where the type of the disease is sub-acute. In certain cases it is of absolutely no value; in others it has proved a failure because an insufficient quantity was given; and in still others it has seemed to aggravate and increase the spasms.†

Knecht, in two very interesting articles in *Schmidt's Jahrbucher* for 1879, collected 389 cases of tetanus, both traumatic and idiopathic, treated in different ways, and carefully tabulated and analyzed them. As a result of this analysis, he gives the following table of percentages:

* *Gaz. des Hôpitaux*, 1874, pp. 516–519.

† H. P. Symonds (*Lancet*) *Medical Gazette*, Feb. 21, 1880.

| No. Cases. | Treatment. | Recovered. | Died. | Mortality. |
|---|---|---|---|---|
| 58 | Surgical.... ........... | 30 | 28 | 48 per cent. |
| 51 | Curare .................. | 26 | 25 | 49 per cent. |
| 60 | Calabar ................. | 33 | 27 | 45 per cent. |
| 134 | Chloral.................. | 79 | 55 | 41 per cent. |
| 63 | Various.. ............... | 32 | 31 | 49 per cent. |

"To the 134 cases treated with chloral may yet be added twenty-three other cases in which this remedy was used in conjunction with other agents. Of these twenty-three cases, nineteen recovered and four died—the mortality of the entire number treated with chloral being thus lowered to thirty-seven per cent.

"The surgical treatment instituted in the fifty-eight cases consisted in amputations, stretching of nerves, etc."

To Knecht's statement that "to the 134 cases treated with chloral may be added twenty-three other cases in which the remedy was used in conjunction with other agents, etc., etc.," I take exception, for if we are to judge of the efficacy of chloral in this disease, we should study only those cases where chloral alone was used. Indeed, many of the cases given in Knecht's table as treated chiefly by chloral, were also treated with chloral and morphine, chloral and the bromide of potassium, chloral and chloroform, etc. In making up the table of the cases which I have collected, I have rearranged some of Knecht's cases, putting them in the second table. I have been able to collect 228 cases, treated by chloral alone, of which 134 recovered and 94 died.

### TABLE I.

| Name of Reporter. | Journal Reference. | Age and Sex. | Cause. | Date after Injury. | Result. | Kind. | Treatment. |
|---|---|---|---|---|---|---|---|
| 1 Marcopoulos | L'Union, 29, 1873. | Young man | Shot in hand | 15 | R. | | Chloral. |
| 2 Bensasson | Do 95, 1871 | M., 13 | Traumatic | | R. | | Chloral. |
| 3 Dorigo | Do do | M., 13 | Traumatic | 20 | R. | | Chloral. |
| 4 Grandisso—Silvestri | Do do | F., 8 | Traumatic | 8 | R. | | Chloral. |
| 5 Schutzenberger | Do do | M., 22 | Cold | | R. | | Chloral. |
| 6–12 Wiederhofer | Do do | Infants | Trismus Nascent | | R. | | Chloral. |
| 13 Runge | Berlin Klin. Woch., vii, 39, 1870 | Adult | Traumatic | 7 | R. | | Chloral. |
| 14 Panthel | Deutscher Klin., 3, 1874. | M., 17 | Traumatic | 5 | R. | | Chloral. |
| 15 Bouchut | Gaz. des Hôpitaux 46–47, 1873 | F., 12 | Traumatic | 21 | D. | | Chloral. |
| 16 Croft | Gaz. de Paris, 51, 1871 | M., adult | Traumatic | 12 | R. | | Chloral. |
| 17 Culver | The Clinic, Aug., 1873 | M., 7 | Traumatic | 7 | D. | | Chloral. |
| 18 Croft | Gaz. de Paris, 51, 1871 | M., 9 | Traumatic | 6 | R. | | Chloral. |
| 19 Lovegrove | Brit. Med. Jour., Nov. 2, 1872 | M., 19 | Traumatic | 22 | R. | | Chloral. |
| 20 Le Fort | Gaz. des Hôp., 62, 1870 | M., 34 | Traumatic | 8 | D. | | Chloral. |
| 21 Delsol | Gaz. Hebdom. 2 S., xi, 31, 1874 | Soldier | Traumatic (gunshot wound) | 12 | R. | | Chloral. |
| 22 Do | Gaz. Hebdom. 2 S., xi, 31, 1874 | M., 15 | Traumatic | 15 | R. | | Chloral. |
| 23 Do | Gaz. Hebdom. 2 S., xi, 31, 1874 | Soldier | Traumatic (gunshot wound) | 10 | D. | | Chloral. |
| 24 Do | Gaz. Hebdom. 2 S., xi, 31, 1874 | M., 24 | Traumatic | 11 | R. | | Chloral. |
| 25 Blin | Bull. Gén. de Thérap., 86, p. 549, June 30, 1874 | M., 22 | Traumatic | 14 | D. | | Chloral. |

| | | | | | | | |
|---|---|---|---|---|---|---|---|
| 26 Do | Bull. Gén. de Thérap., 86, p. 549, June 30, 1874. | Soldier | Traumatic | 18 | D. | Acute | Chloral. |
| 27 Do | Bull. Gén. de Thérap., 86, p. 549, June 30, 1874. | F., adult | Traumatic | 2 | D. | Acute | Chloral. |
| 28 Do | Bull. Gén. de Thérap., 86, p. 549, June 30, 1874. | Young man | Traumatic | 8 | R. | Acute | Chloral. |
| 29 Bourdy | Bull. Gén. de Thérap., 86, p. 549, June 30, 1874. | M., 29 | Traumatic | 3 | R. | | Chloral. |
| 30 Bavdon | Bull. Gén. de Thérap., 86, p. 549, June 30, 1874. | M., 13 | Traumatic | 2 | D. | | Chloral. |
| 31 Do | Bull. Gén. de Thérap., 86, p. 549, June 30, 1874. | F., 64 | Traumatic | 7 | D. | | Chloral. |
| 32 Do | Bull. Gén. de Thérap., 86, p. 549, June 30, 1874. | M., 23 | Traumatic | 8 | D. | | Chloral. |
| 33 Mascaro, J. B. | Gaz. de Paris, 35, 1874. | M., negro, 30. | Traumatic | 3 | R. | | Chloral. |
| 34 Do | Do do | F., 5. | Traumatic | 7 | R. | | Chloral. |
| 35 Chauvel | Record de Med. Milit, 38, xxx, p. 388, July and Aug., 1874 | M., 21 | Traumatic (gunshot). | 18 | R. | | Chloral. |
| 36 Liégeois | Gaz. des Hôp., 62, 1871. | Young man | Traumatic (shot) | 18 | R. | | Chloral. |
| 37 Lavo, G. | Annal. Universal, ccxix, p. 305, fol. 72. Lancet, Mar. 30, 1872 | M., 26 | Traumatic | 14 | R. | | Chloral and warm baths. |
| 38 Do | Annal. Universal, ccxix, p. 305, fol. 72. Lancet, Mar. 30, 1872 | M., 42 | Traumatic | 25 | R. | | Chloral and warm baths. |
| 39 Do | Annal. Universal, ccxix, p. 305, fol. 72. Lancet, Mar. 30, 1872 | M., 22 | Traumatic | 14 | R. | | Chloral and warm baths. |
| 40 Huttenbrenner | Jahrb. für Kinderheilk, n. f. vii, 1, p. 34, 1873 | Child 13 days. | Suppuration of navel | | R. | | Chloral dissolved in mother's milk. |
| 41 Do | Jahrb. für Kinderheilk, n. t. vii, 1, p. 34, 1873. | Child 21 days. | | 8 | R. | | Chloral after 20th day. |

TABLE I.—*Continued.*

| NAME OF REPORTER. | JOURNAL REFERENCE. | AGE AND SEX. | CAUSE. | DATE AFTER INJURY. | RESULT. | KIND. | TREATMENT. |
|---|---|---|---|---|---|---|---|
| 42 Huttenbrenner | Jahrb. für Kinderheilk, n. t. vii, 1, p. 34, 1873. | Infant ....... | .................... | ...... ... | D. | ............ | Chloral. |
| 43–47 Giraldes... | Gaz. des Hôp., 62, 1871.. | Soldiers ...... | Traumatic.......... | ......... | D. | ............ | Chloral. |
| 48 Blot.......... | Do do .. | Soldier...... | Traumatic.......... | ......... | D. | ............ | Chloral. |
| 49–50 Boinet..... | Do 21, 1874.. | Males ........ | Traumatic.......... | ......... | R. | .......... | Chloral. |
| 51 Do ..... | Do do .. | M........... | Traumatic.......... | ......... | D. | ............ | Chloral. |
| 52 Do .... | Do do .. | M.... ...... | Traumatic.......... | ......... | D. | ........... | Chloral. Died from hæmorrhage. |
| 53–57 Verneuil... | Do do .. | Soldiers ...... | Traumatic.......... | ......... | R. | ............ | Chloral. |
| 58 Ferrier....... | Do 112, 1874. | M., 61........ | Traumatic.......... | 9 | D. | ............ | Chloral. |
| 59 Richelot...... | Do 65, 1874.. | M. .......... | Traumatic.......... | 2 | R. | ............ | Chloral. |
| 60 Do ...... | Do do .. | M., 36.... ... | Traumatic.......... | 10 | R. | ...... .... | Chloral. |
| 61 Baizeau .... | Do 88, 1874.. | Soldier....... | Traumatic (shot).... | 6 | D. | ........... | Chloral. |
| 62 Blain..... ... | Do 94, 1874.. | F. .......... | Traumatic.. .. .... | 4 | D. | ............ | Chloral. |
| 63 Do. ......... | Do do .. | M., 24..... ... | Traumatic.......... | 8 | R. | ............ | Chloral. |
| 64 Guyon........ | L'Union, 60, 1870....... | F., 29......... | Traumatic....... ... | 11 | D. | ............ | Chloral. |
| 65 Duplay ...... | Do 85, 1874 ...... | M., 25........ | Traumatic.......... | 3 | D. | ............ | Chloral. |
| 66 Coryllos (Patras) ....... | Do 96, 1874....... | M., 46........ | Traumatic.......... | 5 | R. | ............ | Chloral. |
| 67 Coryllos (Patras) ....... | Do do ... ... | F., 40......... | Traumatic.......... | 30 | R. | .......... | Chloral. |
| 68 Nicholls (West Indies)..... | Med. Times & Gaz.. May 27, 1876..... ....... | M., negro, 46 . | Traumatic.......... | 14 | R. | ........... | Chloral. |
| 69 Nicholls (West Indies)..... | Med. Times & Gaz., May 27, 186.............. | M., negro, 20 . | Traumatic.......... | 7 | R. | .......... | Chloral. |
| 70 Köhler ....... | Deutsche Milit. Arztl. Ztschr., ii, 5, p. 267, 1873 | Soldier...... | Traumatic (shot).... | 8 | R. | ............ | Chloral. |

| | | | | | | |
|---|---|---|---|---|---|---|
| 71 Marin | Gaz. Lomb. xxxv, 7, 1875 | M., 12 | Traumatic | 9 | R. | Chloral. |
| 72 Carrick | The Clinic, 13, 1874 | M., 12 | Traumatic | 6 | R. | Chloral. |
| 73 Oré (Bordeaux) | Gaz. de Paris, 21, 1874 | M. | Traumatic | | R. | Chloral (intravenous). |
| 74 Cruveilher | Gaz. des Hôp. 49, 1874 | M., 33 | Traumatic | 15 | D. | Chloral (intravenous). |
| 75 Labbé | Gaz. de Paris, 21, 1874 | M., 29 | Gangrene | 28 | D. | Chloral (intravenous). |
| 76 Tillaux | L'Union, 101, 1874 | F., 46 | Traumatic | 10 | D. | Chloral (intravenous). |
| 77 Lannelongue | Gaz. des Hôp., 125, 1874. | M., 13 | Traumatic | 8 | D. | Chloral (intravenous). |
| 78 Davis, G. W. | Letter to the author | Young man | | | R. | Chloral. |
| 79 Rafferty | Phila. M. & S. Reporter, 24, 1874 | M., 40 | Idiopathic (cold and wet) | 2 | D. | Chloral. |
| 80 Hansen | Dorp. Med. Ztschr., v, 8, p. 290, 1874 | M., 40 | Idiopathic (cold and wet) | 1 | R. | Chloral. |
| 81 Wauthy | Jour. de Broux, Dec., 1875, p. 522 | | Operation | 11 | D. | Chloral. |
| 82 Do | Jour. de Broux, Dec., 1875, p. 522 | | Traumatic | 10 | R. | Chloral. |
| 83 Boucque | Presse Med., 1876 | | Traumatic | 16 | D. | Chloral. |
| 84 Cane, Leonard | Lancet, April, 1876 | M., 28 | Idiopathic | 21 | R. | Chloral. |
| 87 Agelastos | Gaz. de Paris, 45, 1876 | | From cold | | R. | Chloral. |
| 88 Do | Do do | | From cold | | R. | Chloral. |
| 89 Tueffard, L. | L'Union, 151, 1876 | | Traumatic | 1 | R. | Chloral. |
| 90 Faulkner, Wm | Phila. M. & S. Reporter, Nov., 1876 | | Traumatic | 12 | R. | Chloral. |
| 91 Bournet | L'Union, 75, 1876 | | From cold | 2 | R. | Chloral. |
| 92 Sourier | Gaz. des Hôp., 46, 1876 | | Traumatic | 17 | D. | Chloral. |
| 93 Durand, Cesar. | Gaz. Hebdom | | Traumatic | 14 | R. | Chloral. |
| 94 Marchioneschi | Lo Sperimentale, Feb., 1876 | | Traumatic | 8 | D. | Chloral. |
| 95 Kocher | Schweiz-Corr. Blat, vi 17, 1876 | M., 45 | Idiopathic | 17 | D. | Chloral. Refused to take after few days. |
| 96 Dyer | Bost. M. & S. Jour., Mar. 1877, p. 306 | | Excoriation and cold | 8 | D. | Chloral. Tetanus nearly well. Death attributed to pneumonia, which had set in. |

TABLE I.—*Continued.*

| Name of Reporter. | Journal Reference. | Age and Sex. | Cause. | Date after Injury. | Result. | Kind. | Treatment. |
|---|---|---|---|---|---|---|---|
| 97 Roberts, J. B.. | Am. Jour. Med. Sci., Oct., 1877............ | M., 19 ....... | Traumatic (gunshot). | ......... | R. | ......... .. | Chloral. |
| 98 Do | Am. Jour. Med. Sci., Oct., 1877......... .. | M., 5......... | Burns .............. | 7 | D. | Acute ...... | Chloral. |
| 99 Do | Am. Jour. Med. Sci., Oct., 1877............ | M , 35... .... | Traumatic.......... | 4 | D. | Acute ...... | Chloral. |
| 100 Do | Am. Jour. Med. Sci., Oct., 1877............ | M., 45 ....... | Traumatic.. ...... | ......... | R. | Acute ...... | Chloral. |
| 101 Do | Am. Jour. Med. Sci., Oct., 1877............ | M., 20........ | Traumatic.......... | ......... | R. | Sub-acute ... | Chloral. |
| 102 Cargill, J.... | Lancet, Aug., 1877...... | ............. | Traumatic.......... | ......... | R. | ........... | Chloral. |
| 103 Do ... | Do do ...... | ............. | Suppurative stage of variola .......... | 15 | R. | Sub-acute ... | Chloral. |
| 104 Beaumetz —Dujardin.. | Bull. Thérap., Sep., 1877 | M., 27 ....... | From cold........ | Same day | D. | ........... | Chloral. Rupture of heart. |
| 105 Alpago—Novello ...... | Annal. Univ., Nov., 1877 | ............. | Traumatic.......... | ......... | R. | Sub-acute ... | Chloral. |
| 106 Alpago—Novello ...... | Do do | M., adult..... | Traumatic.......... | ......... | R. | ........... | Chloral. |
| 107 Alpago—Novello ...... | Do do | M., adult..... | Traumatic.......... | ......... | R. | ....... .... | Chloral. |
| 108 Alpago—Novello ...... | Do do | ............. | Traumatic.......... | ......... | D. | ........... | Chloral. |
| 109 Alpago—Novello ...... | Do do | ............. | Traumatic.......... | ......... | R. | ........... | Chloral. |
| 110 Pinoli....... | Do do | ........... | Not stated.......... | ......... | R. | .... .... | Chloral and warm baths. |
| 111 Verneuil..... | Gaz. Hebdom., 1876..... | M., 47........ | Frozen feet ..... ... | 13 | D. | ........... | Chloral. |

| | | | | | | | | |
|---|---|---|---|---|---|---|---|---|
| 112 | Do | Do do | M., 15 | Traumatic | 10 | D. | | Chloral. |
| 113 | Fitzgibbon, H | Dublin Jour., Mar., 1877 | M., 12 | Traumatic | 5 | D. | | Chloral. Spasm apparently aggravated by chloral. |
| 114 | Duncan, G. C. | Canada M. & S. Jour., June, 1877 | | Traumatic | 8 | R. | | Chloral. |
| 115 | Lawson, A. | Lancet, Feb. 16, 1878 | M., 9. | Traumatic | 9 | R. | | Chloral. |
| 116 | Krause, Robt. | Inaug. Dissertation, Breslau, 1878 | M., 40 | Frozen toes | | D. | | Chloral. |
| 117 | Do | Inaug. Dissertation, Breslau, 1878 | F., 28 | Frozen toes | | D. | | Chloral. |
| 118 | Medini, Luigi | Lo Sperimentale, 1878, p. 454 | M., 31 | Traumatic | 16 | R. | | Chloral. |
| 119 | Lorenzutti, Lorenzo | Annal. Univ., Nov., 1876 | | From cold | 3 | R. | | Chloral. |
| 120 | Luzatto B. | Lo Sperimentale, 1876 | F., 30 | Traumatic | Few days. | R. | | Chloral. |
| 121 | Watson, Eben | Lancet, 1870 | | Traumatic | 8 | D. | | Chloral. |
| 122 | Nankwell | Do Mar., 1878 | | Traumatic | | D. | | Chloral. |
| 123 | Do | Do do | | Traumatic | 8 | D. | | Chloral. |
| 124 | Ribell | Annal. de Gynécol., 1875 | F. | Post-partum | 63 | R. | Acute | Chloral (hypodermically). |
| 125 | Dufour | Bull. Gén. de Thérap., 1870, T. 78, p. 566 | | Traumatic | 8 | R. | | Chloral. |
| 126 | Laurens | Practitioner, 1877, p. 142 | Woman | Traumatic | 8 | R. | | Chloral. Continuous sleep for eight days. |
| 127 | Lavo, G. | Annal. Universal, Feb., 1872 | | Traumatic | 25 | R. | | Chloral and warm baths. |
| 128 | Maxwell, Allison | Am. Practitioner, Jan., 1880 | M., 9 | Traumatic | 13 | R. | Sub-acute | Chloral. |
| 129 | | Gaz. des Hôp., 1870 | | Traumatic | 22 | R. | | Chloral. |
| 130 | Johnson | Jour. Psycholog. Med., 1871 | M., 13 | Traumatic | 9 | R. | | Chloral. |
| 131 | Pugliere | Practitioner, 1875, p. 299 | | Traumatic | | R. | Acute | Chloral. |
| 132 | Ballantyne | Lancet, 1870 (October) | M., 34 | Traumatic | 12 | R. | Acute | Chloral, and thorn removed from wound. |

TABLE I.—*Continued.*

| Name of Reporter. | Journal Reference. | Age and Sex. | Cause. | Date after Injury. | Result. | Kind. | Treatment. |
|---|---|---|---|---|---|---|---|
| 133 Cruveilher .. | Le Progrès Méd., July, 1875 ................ | .............. | Unknown.......... | ......... | R. | ............ | Chloral. |
| 134 Deu, A...... | Bull. Gén. de Thérap., 1875 ................ | ... ... ....... | Traumatic. ........ | 9 | D. | .... ... ..... | Chloral. |
| 135 Lawrence, A G | Lancet, June, 1871...... | F. ........... | Traumatic.......... | 1 | R. | Sub-acute ... | Chloral. |
| 136 Thompson, G. | Do April, 1871..... | M., 20 ...... | Idiopathic.......... | ......... | R. | ............ | Chloral. |
| 137 Eager....... | Do Mar., 1871...... | M., 23........ | Traumatic.......... | 19 | D. | ........... | Chloral. |
| 138 Tyrrell...... | Do May, 1871...... | M., 23.... | Traumatic......... | 7 | D. | ........... | Chloral. |
| 139 Tait, Lawson. | Do Jan., 1871...... | M., 32....... | Traumatic.......... | 6 | D. | ....... ... | Chloral. |
| 140 Gay, G. W... | Boston M. & S. Jour., Aug., 1876... ....... | .............. | Traumatic.......... | 10 | D. | ... ........ | Chloral. |
| 141 Madden .... | Dublin Jour., 1874, p. 583 | F. ........... | Puerperal.......... | ......... | R. | ........ .... | Chloral. |
| 142 Vilaplana.... | Lond. Med. Record, Jan., 1877 ............... | .............. | Traumatic.......... | 20 | R. | ........ .... | Chloral. |
| 143 Salter, J. H.. | Practitioner, Dec., 1876. | M., 38........ | Traumatic...... ... | 11 | R. | ............ | Chloral (hypodermically). |
| 144 ...... ... ... | Bull. Gén. de Thérap., 1870, p. 477.......... | .............. | Traumatic.......... | ......... | D. | ......... ... | Chloral. |
| 145 ............ | Bull. Gén. de Thérap., 1870, p. 477.......... | .............. | Traumatic.......... | ......... | D. | ....... .... | Chloral. |
| 146 Monette, G. N. | Med. Brief, Sept., 1877; Am. Jour. Med. Sci., Oct., 1875... ....... | F., 2 yrs...... | From vaccinat'n that progressed badly.. | 27 | R. | Sub-acute ... | Chloral. |
| 147 Gueniot & Gamiez ... | Med. News & Library, Nov. 1877. .......... | .............. | Traumatic.......... | ...... ... | R. | ........... | Chloral. |
| 148 Dunster, E. S. | N. Y. Med. Jour., 1871, p. 748........ ...... | ............. | Not stated.......... | ....... . | D. | ............. | Chloral. |

| | | | | | | | |
|---|---|---|---|---|---|---|---|
| 149 Paget | N. Y. Med. Jour, 1871, p. 118 | | Not stated | | D. | | Chloral. |
| 150 Broadbent | N. Y. Med Jour., 1871, p. 118 | | Idiopathic | | D. | | Chloral. |
| 151 Nichol, W. L. | N. Y. Med. Jour., 1870, p. 455 | M., mulatto, 14 | Traumatic | Few days. | R. | | Chloral, after opium and bromide had failed. |
| 152 Liebreich, O. | Berlin Klin. Woch., Oct. 24, 1870 | | Idiopathic | | R. | | Chloral. |
| 153 Bellevue Hospital | N. Y. Med. Record, Jan., 1871 | M., 9 | Traumatic | 9 | D. | Acute | Chloral. |
| 154 Bellevue Hospital | N. Y. Med. Record, Jan. 1871 | M., 28 | Traumatic | 19 | R. | Acute | Chloral. |
| 155 MacDonald, A | Edin. Med. Jour., June, 1875 | F. | Puerperal | | D. | Very severe. | Chloral. |
| 156 Knox, D. J. S. | Edin. Med. Jour., Jan., 1872 | M., 17 | Traumatic | 28 | R. | | Chloral. |
| 157 Laraux, Dubreil & Onimus. | Gaz. des Hôp., 68, 1870. | Man | Traumatic | 17 | R. | Acute | Chloral and continuous current. |
| 158 Stutel | Do 1878, 942. | | Traumatic | 15 | R. | | Chloral. |
| 159 Wirth, R. | N. Y. Med. Jour., 1870, p. 419 | Man | Traumatic | 14 | R. | Sub-acute | Chloral, after morphine and opium had failed to affect. |
| 160 Watson, Spencer | Lancet, Oct., 1870 | F., 41 | Traumatic | 21 | R. | Sub-acute | Chloral. |
| 161 Langier (quoted by Labbé). | Bull. Gén. de Thérap., 1870, p. 330 | | Traumatic | | D. | | Chloral. |
| 162 Bouchut | Bull. Gén. de Thérap., 1870, p. 330 | | Idiopathic | | D. | | Chloral. |
| 163 Demarquay | Bull. Gén. de Thérap., 1870, p. 330 | | Traumatic | | D. | | Chloral. |

Table I.—*Continued.*

| Name of Reporter. | Journal Reference. | Age and Sex. | Cause. | Date after Injury. | Result. | Kind. | Treatment. |
|---|---|---|---|---|---|---|---|
| 164 Limousin | Bull. Gén. de Thérap., 1870, p. 330 | | Traumatic | | D. | | Chloral. |
| 165 Liebreich | Bull. Gén. de Thérap., 1870, p. 330 | | Traumatic | | D. | | Chloral. |
| 166 Langenbeck | Bull. Gén. de Thérap., 1870, p. 330 | | Traumatic | | R. | | Chloral. |
| 167 Dufour | Gaz. des Hôp., 65, 1870 | | | | D. | | Chloral. |
| 168 Do | Do do | | | | D. | | Chloral. |
| 169 Do | Do 78, p. 566, 1870 | Man | Traumatic | 8 | R. | | Chloral. |
| 170 Bleynie | Lancet, July 15, 1876 | M., 50 | Traumatic | 12 | R. | Acute | Chloral. |
| 171 Bigelow, H. R. | Letter to author | M., 36 | Idiopathic | | D. | | Chloral. |
| 172 Johnson, Geo. | Jour. Psycholog. Med. 1871, p. 150 | | Traumatic | 21 | R. | | Chloral. |
| 173 Geens | Jour. de Broux, Dec., 1871 | | Traumatic | | R. | | Chloral. |
| 174 Weber, H. | Med. Times & Gaz., May 11, 1872 | M., 35 | Idiopathic | | R. | Sub-acute | Chloral, after use of morphia for two days. |
| 175 Beck, J. R. | St. Louis M. & S. Jour., June, 1872 | | Traumatic | 7 | R. | Acute | Chloral. |
| 176 Cluness | Pacific M. & S. Jour., Apr., 1871 | | Traumatic | | R. | | Chloral. |
| 177 Tay | Brit. Med. Jour., Apr., 1870 | | Traumatic | | D. | | Chloral. |
| 178 Peter, Preston | Am. Practitioner, Sept., 1870 | | | | R. | | Chloral. |
| 179 Truckwell & Winckfield. | Lancet, Aug. 23, 1879 | M., 25 | Idiopathic | | D. | Sub-acute | Chloral. |

| | | | | | | | |
|---|---|---|---|---|---|---|---|
| 180 Peele, Edward | Dublin Jour., Apr., 1879 | M., 6 | Traumatic | 10 | R. | Sub-acute | Chloral. |
| 181 Macnamara | Practitioner, 1871 | | Traumatic | | D. | | Chloral. |
| 182 Do | Do | | Traumatic | | D. | | Chloral. |
| 183 Do | Do | | Traumatic | | D. | | Chloral. |
| 184 Do | Do | | Traumatic | | D. | | Chloral. |
| 185 Do | Do | | Traumatic | | D. | | Chloral. |
| 186 Do | Do | | Traumatic | | D. | | Chloral. |
| 187 Do | Do | | Traumatic | | R. | | Chloral. |
| 188 Do | Do | | Idiopathic | | R. | | Chloral. |
| 189 Do | Do | | Idiopathic | | R. | | Chloral. |
| 190 Do | Do | | Idiopathic | | R. | | Chloral. |
| 191 Kilpatrick | Letter to the author | M., 14 | Traumatic | 11 | R. | | Chloral. |
| 192 Garneis (Beck) | St. Louis M. & S. Jour., June, 1872 | | Traumatic | | R. | | Chloral. |
| 193 Do | St. Louis M. & S. Jour., June, 1872 | | Traumatic | | R. | | Chloral. |
| 194 Prewitt | St. Louis M. & S. Jour., Oct., 1876 | M., 7 | Traumatic | 7 | R. | | Chloral. |
| 195 White, Isa. H. | Letter to author | M., 25 | Traumatic | | R. | Acute | Chloral, after calabar bean had failed. |
| 196 Byrce, C. A. | Do | M., 52 | Traumatic | | R. | Sub-acute | Chloral and milk enemata. |
| 197 Miller, J. K. | Phila. M. & S. Reporter, Dec., 1877 | M., 8 | Traumatic | 5 | R. | | Chloral, after belladonna and calabar bean. |
| 198 Delmont, F. | Letter to author | M., 6 | Traumatic | 10 | R. | Acute | Chloral. |
| 199 Corme, Alf. | Lancet, Oct. 30, 1875 | M., 19 | Poisoned arrow | 6 | D. | Acute | Chloral. Morphia once or twice. |
| 200 Boislinière | St. Louis M. & S. Jour., Oct, 1876 | Infant | Trismus nascentium | | R. | | Chloral. |
| 201 | Gaz. des Hôp., 1877, p. 741 | Woman | After removal of breast | 22 | R. | | Chloral. |
| 202 Arton, J. H. | Letter to author | M., negro, 16 | Traumatic | 10 | R. | Sub-acute | Chloral. |

TABLE I.—*Continued.*

| Name of Reporter. | Journal Reference. | Age and Sex. | Cause. | Date after Injury. | Result. | Kind. | Treatment. |
|---|---|---|---|---|---|---|---|
| 203 Arton, J. H... | Letter to author........ | M., 10 ....... | Traumatic.. .. .... | 10 | D. | Acute ...... | Chloral. Seen only 36 hours before death. Had previously been treated by valerian. |
| 204 Do .. | Do | M., 12........ | Traumatic...... ... | 10 | D. | Acute ...... | Chloral. |
| 205 Roof, Stephen | Do ......... | F., 9 days .... | Trismus nascentium. | ......... | D. | ............ | Chloral. |
| 206 Chauvel..... | Bull. Gén. de Thérap., vol. 86, p. 422........ | .............. | ..................... | ......... | D. | ............ | Chloral. |
| 207 Do ..... | Bull. Gén. de Thérap., vol. 86, p. 422........ | .............. | ..................... | ......... | D. | ............ | Chloral. |
| 208 Rasia, Dom.. | Gaz. Med. Italiano, prov. Venetta, Dec. 25, 1875. | .............. | Traumatic........... | ......... | R. | ............ | Chloral. |
| 209 F. Delmont.. | San Buenuventa, Cal. By letter to author....... | .............. | Traumatic........... | ......... | R. | ............ | Chloral. |
| 210 Linde, G. de la | El Genio Medico Quirurgico, Sept. 15, 1875... | .............. | Traumatic ........ | ......... | R. | ............ | Chloral. |
| 211 Do | El Genio Medico Quirurgico, Sept. 15, 1875.. | .............. | Traumatic....... .. | ......... | R. | ............ | Chloral. |
| 212 Tillaux...... | Gaz. des Hôpitaux, 1876, p. 211............... | .............. | ..................... | ......... | R. | ............ | Chloral. |
| 213 P. Kretschmar | Personal communication | M., 17 ....... | ..................... | ......... | D. | ............ | Chloral. |
| 214 Do | Do do | Infant....... | Trismus nascentium. | ......... | D. | ............ | Chloral. |
| 215 H. W. Boyd (Chicago, Ill.) | Letter to the author..... | ......... | ..................... | .. ..... | R. | ............ | Chloral. |
| 216 Mr. Birkett.. | Taylor, Guy's Hosp. Reports, 1878, p. 339.... | M., 21........ | Traumatic.......... | 1 | R. | Chronic .... | Chloral. |
| 217 Mr. Hilton... | Taylor, Guy's Hosp. Reports, 1878, p. 339.... | M........... | Traumatic.......... | 11 | D. | ............ | Chloral |

| | | | | | | | | |
|---|---|---|---|---|---|---|---|---|
| 218 | Dr. Moxon... | Taylor, Guy's Hosp. Reports, 1878, p. 349.... | F., 43. ....... | Idiopathic........ | ......... | D. | ... ........ | Chloral; ten drachms in five days. |
| 219 | Dr. Forster.. | Taylor, Guy's Hosp. Reports, 1878, p. 339.... | M., 16........ | Traumatic.......... | 21 | D. | ............ | Chloral. |
| 220 | Do .. | Taylor, Guy's Hosp. Reports, 1878, p. 339.... | M............ | Traumatic......... | 9 | D. | .... ....... | Chloral. |
| 221 | Mr. Bryant.. | Taylor, Guy's Hosp. Reports, 1878, p, 339.... | M., 43........ | Traumatic.......... | 14 | D. | ............ | Chloral and tracheotomy. |
| 222 | Mr. Forster.. | Taylor, Guy's Hosp. Reports, 1878, p. 336.... | M., 28....... | Traumatic.......... | 13 | D. | ............ | Chloral. (Autopsy showed purulent mucus in bronchi.) |
| 223 | Dr. Wilks ... | Taylor, Guy's Hosp. Reports, 1878, p. 336 ... | F., 35......... | Idiopathic....... ... | 1 | D. | ............ | Chloral. (Bloody sweat.) |
| 224 | Mr. Durham. | Taylor, Guy's Hosp. Reports, 1878, p. 339.... | F., 14......... | Traumatic ........ | 9 | D. | ............ | Chloral. |
| 225 | A. Maxwell, (Indianapolis) | Letter to the author.... | ............. | Traumatic. ........ | ......... | R. | Acute ...... | Chloral. |
| 226 | R.L.Pinching (San Francisco) | Do do ..... | ............. | ................... | ......... | D. | ............ | Chloral. |
| 227 | R.L.Pinching (San Francisco) | Do do ..... | ............. | ................... | ......... | D. | ............ | Chloral. |
| 228 | L. C. Herrick (Woodstock,O.) | Do do ..... | ............. | ................... | ......... | R. | ............ | Chloral. |

| | | | |
|---|---|---|---|
| Total cases ...228 | Males ..............119 | Traumatic.........103 | |
| | | Idiopathic......... 10 | |
| | Females........... 22 | Traumatic......... 17 | |
| | | Idiopathic......... 2 | |
| | | Puerperal......... 3 | |
| | Not stated and infants ......... 87 | Tetanus, infant... 12 | |
| | 228 | | |

| | | | |
|---|---|---|---|
| Total cases....228 | Traumatic.........175 | Males ......103 | 175 |
| | | Females.... 17 | |
| | | Not stated..108 | |
| | Idiopathic......... 21 | Males ...... 10 | 21 |
| | | Females.... 2 | |
| | | Not stated.. 9 | |
| | Infantile tetanus.. 12 | | |
| | Puerperal tetanus 3 | | |
| | 211 | | |
| | Not stated......... 17 | | |
| | 228 | | |

| | | | |
|---|---|---|---|
| Recoveries ...134 | | | |
| Deaths ........ 94 | Males .............. 45 | Traumatic. 41 | 45 |
| | | Idiopathic.. 4 | |
| | Females........... 11 | Traumatic. 8 | 11 |
| | | Idiopathic. 2 | |
| | | Puerperal.. 1 | |
| | Infants ............ 3 | | |
| | Not stated......... 35 | | |
| | 94 | | |

In the second table, which I here give, various remedies were used, chloral, however, always entering into and playing a prominent part in the treatment. There are 98 cases in all, with 64 recoveries and 34 deaths, really a better showing than is to be had in those cases where chloral alone was used.

From the facts shown in Table II it can be plainly seen that success under the chloral treatment alone is not so great as that under a mixed treatment into which chloral largely enters, and consequently that this drug is by no means a specific in this disease ; that males are more likely to be attacked than females, and that of the females so affected a larger percentage than of the males die.

It is a noticeable fact, also, that the cases of tetanus occurring in hospital yield a very large percentage of deaths under any form of treatment. I am of the opinion that this is due to three things: 1st. That hospital patients are, as a rule, but poorly nourished. 2d. That these cases are usually of the acute type. 3d. That in many instances they are not brought into a hospital until the disease has been under way for from one to four days.

Tetanus, during war, has always yielded a very large percentage of deaths. McLeod,* "Notes on the Surgery of the War in Crimea," relates twenty-three cases, in which there were but two recoveries; and Demme* states that eighty-six cases treated in the Italian hospitals during the war of 1859 yielded but six recoveries. These were of course not treated by chloral.

Dr. O'Beirne* (Dublin Hospital Reports) gives 200 cases with no recoveries.

Some of the cases of tetanus occurring in both hospital and private practice, but especially the latter, are undoubtedly hysterical and would recover, sooner or later, under any plan of treatment. Dr. D. Webster Prentiss† gives the following rules for distinguishing true from hysterical tetanus.

In a case reported of the latter condition, he states that the points in the case marking it as hysterical were—

1. It was ushered in by noise in the ears, deafness and blindness.

In true tetanus and strychnia poisoning the senses are rendered preternaturally acute.

2. There was unconsciousness during the paryoxysms.

This never occurs in tetanus and strychnia poisoning, except as an ante-mortem condition.

3. The eyes were closed during the spasms. The eyes stare widely open in the other diseases.

---

* Quoted by E. C. Mann, N. Y. Med. Record, Jan. 16, 1875.

† Braithewaite's Quarterly Epitome, March, 1880.

### TABLE II.

| Name of Reporter. | Journal Reference. | Age and Sex. | Cause. | Date after Injury. | Result. | Kind. | Treatment. |
|---|---|---|---|---|---|---|---|
| 1 Upshur, J. N.. | Virginia Med. Monthly, May, 1880............ | M. (negro) 4.. | Idiopathic.... | .. .. | D. | ...... | Chloral and bromides. |
| 2 Do....... | Virginia Med. Monthly, May, 1880............ | ............. .... | Traumatic.... | 17 | R. | ...... | Chloral, bromides and morphia. |
| 3 Spence ........ | Lancet, April, 1876...... | M., 5[illegible] ......... | Traumatic.... | 30 | R. | ...... | Chloral, potass. bromid., atropia and amputation of leg. |
| 4 Groneman ..... | Lancet, Feb., 1871...... | M., 44......... | Traumatic.... | ...... | R. | ...... | Chloral and morphine. |
| 5 Gay, G. W..... | Boston M. & S. Journal, Oct., 1877............ | F., 21 ........ | Traumatic.... | 24 | R. | ...... | Chloral and potass. bromid. |
| 6 Griffin, H...... | Practitioner, Feb., 1872.. | F., 21 ........ | Traumatic.... | 22 | R. | ...... | Chloral, morphine and galvanism. |
| 7 Boyd, M. A.... | Dublin Jour., 1874, p. 583 | F., adult...... | Puerperal .... | 6 | D. | ...... | Chloral and chloroform. |
| 8 Denton ....... | Practitioner, 1870, p. 370. | Boy.......... | Traumatic .. | ...... | R. | ...... | Chloral and other drugs. |
| 9 Salisbury...... | Chicago Med. Journal, Dec., 1879........... | M., 16......... | Traumatic.... | 10 | R. | ...... | Chloral, potass. bromid., and towards the last, calabar bean. |
| 10 Do. ... ... | Chicago Med. Journal, Dec., 1879............ | M., .... ..... | Traumatic.... | 6 | D. | ..... | Chloral, potass. bromid., and towards the last, calabar bean. |
| 11 Watson, P. E.. | Lancet, Feb., 16, 1878... | F., adult...... | Idiopathic .... | 10 | R. | ...... | Chloral and atropia hypodermically. |
| 12 Cushing, C.... | Pacific M. & S. Journal, March, 1872.......... | M., 5......... | Traumatic.... | 14 | R. | Mild.. | Chloral and calabar bean. |
| 13 Shelley, A. W.. | N. Y. Med. Jour., 1873, p. 262................ | F., 39...... .. | Traumatic .... | 14 | R. | ...... | Chloral and morphia. |
| 14 Maxwell, A... | Am. Practitioner, Jan., 1880................. | ............. .... | Traumatic.... | ...... | R. | Acute. | Chloral, morphia and atropia. |
| 15 Leeds, O. H... | N. Y. Med. Jour., 1875, p. 393 . ............ | F., 14 ........ | Traumatic.... | 15 | R. | ...... | Chloral and potass. bromid. |
| 16 Do ..... | N. Y. Med. Jour., 1875, p. 393 ............... | F., 28....... | Traumatic.... | 10 | R. | S. A.. | Chloral and potass. bromid. |

| | | | | | | | |
|---|---|---|---|---|---|---|---|
| 17 .......... | Annal. Universal; N. Y. Med. Jour., Vol. 25, p. 234.. .......... | .......... | Traumatic.... | ...... | R. | ...... | Chloral and jaborandi. |
| 18 Ogle........ | N. Y. Med. Jour., 1871, p. 117.............. | Boy.......... | Traumatic.... | 3 | R. | ...... | Chloral, belladonna and ice to spine. |
| 19 Curran, J. W.. | Med. Press & Circular, Oct., 1870 ... ...... | .......... | Traumatic.... | .... | D. | ...... | Chloral and belladonna. |
| 20 Molliere...... | Gaz. des Hôp., 1879..... | M., 25........ | Traumatic.... | 14 | R. | Acute. | Symptoms caused after amputation, Chloral, bromide potass., morphia. |
| 21 Royds, Wm... | Lancet, Dec., 1870...... | M., 25........ | Traumatic.... | 10 | D. | ...... | Chloral and calabar been. Death from hemmorrhage from wound from tetanus. |
| 22 Do......... | Do do .... | M., 13........ | Traumatic.... | ...... | R. | ...... | Chloral, chloroform and physostigma. |
| 23 Bryant, Thos.. | Do Nov. 2, 1872 ... | M., 19........ | Traumatic ... | 19 | R. | ..... | Chloral; then chloral and morphia. |
| 24 Martyn....... | Do Nov. 21, 1874... | M., 15........ | Not decided... | ...... | D. | Acute. | Chloral, potass. bromid. and ice to spine. |
| 25 Hornby....... | Do Oct. 19, 1872 ... | M., 32........ | Traumatic.... | 3 | D. | ..... | Chloral, morphia and ice to spine. |
| 26 Do......... | Do do .... | M., 40........ | Traumatic... | Fri. | R. | ...... | Chloral and nicotine. |
| 27 Duncan, A. E. | Cincinnati Med. News.. | F., 19........ | Traumatic.... | 1 | R. | Acute. | Chloral, chloroform during spasms. |
| 28 Hunter, G. G.. | Lancet, Feb. 6, 1875 ... | M., 13........ | Traumatic.... | 10 | R. | ..... | Potass. bromid, cannabis indica and small amt. chloroform. Chloral only as an hypnotic. |
| 29 Hamilton, J. B. | Do Jan. 16, 1875... | M., 25 ....... | Traumatic ... | 14 | D. | ...... | Chloral and cannabis indica. |
| 30 Smith, Spencer | Do May 22, 1875... | M., 27 ....... | Traumatic.... | 15 | R. | S. A.. | Chloral and potass. bromid. |
| 31 Shinkwin, T. C. | Dublin Jour., Aug., 1875. | Young men | Traumatic.... | 13 | R. | Acute. | Chloral (most benefit from) and calabar bean. |
| 32 Southey ...... | Lancet, Oct. 16, 1875.... | M., 20 ....... | Idiopathic.... | 2 | R. | ..... | Chloral, finally abandoned for potass. bromid and conium. |
| 33 Mackay, W. B. | Brit. Med. Jour., 1880... | M., 24.... ... | T. (?) ...... | 14 | R. | Acute. | Chloral and belladonna. |
| 34 .. ... ....... | Hospital Gazette...... | ........ ..... | .............. | ...... | R. | ..... | Chloral and potass. bromide (enemata). |
| 35 Roberts, H. P. | Lancet, May 20, 1876... | ............. | Traumatic.... | 4 | D. | ...... | Chloral (of no service), chloroform and morphia. |
| 36 Do...... | Do do ... | ............. | Traumatic.... | 8 | D. | ...... | Chloral, chloroform and morphia. |

TABLE II.—*Continued.*

| Name of Reporter. | Journal Reference. | Age and Sex. | Cause. | Date after Injury. | Result. | Kind. | Treatment. |
|---|---|---|---|---|---|---|---|
| 37 Baker........ | Lancet, April 15, 1876.. | M., 29........ | Traumatic.... | 13 | D. | ...... | Potass. bromid, morphia and hyoscyamin first 3 days. Then chloral. Nerve was decided also. |
| 38 Do......... | Do do .. | F., 29 ........ | Traumatic.... | 11 | D. | ...... | Chloral and chloroform. Finger amputated. |
| 39 Benton, Sam'l.. | Lancet, Nov. 11, 1876 .. | M., 31 ....... | Idiopathic .... | ...... | D. | ...... | Chloral, morphia and potass. bromid. |
| 40 Do..... | Do March 24, 1874.. | ............. | Traumatic.... | 9 | R. | ...... | Chloral, potass. bromid and tr. opii. |
| 41 Boon, A. T.... | Do July 28, 1877... | ............. | Idiopathic.... | ...... | R. | S. A.. | Chloral and calabar bean. |
| 42 Duncan, T. G.. | Am. Prac., Aug., 1870... | ............. | ............... | ...... | R. | ...... | Chloral and calabar bean. |
| 43 Kirchoff...... | Berlin Klin. Woch, 1879, No. 25............... | F., 52 ........ | Traumatic.... | 6 | D. | ...... | Chloral and potass. bromid in large doses. Thorn removed. |
| 44 Kinsman, D. N. | (Letter to the Author).. | F., 12 ....... | Traumatic.... | 3 | D. | Acute. | Chloral alone at first. Subsequently morphia, atropia, calabar bean and chloroform. |
| 45 Easley, E. T... | Do do .. | M., 19 ....... | Traumatic.... | 11 | R. | Acute. | Chloral, morphia hypodermically. |
| 46 Do...... | Do do .. | M., 14 ....... | Traumatic.... | 7 | R. | S. A.. | Chloral, morphia hypodermically. |
| 47 Squier, Jno. B. | Do do .. | M., 60........ | Traumatic.... | 10 | D. | A .... | Aconite, chloral and morphia. |
| 48 Laveran ...... | Archiv. de Physiol., Oct., 1877 ................ | M., 11½...... | Traumatic .... | 12 | D. | ..... | Chloral and morphia. |
| 49 Rizzoli. ..... | L' Union, 1873, p. 247... | M., 19........ | Traumatic.... | 9 | R. | ...... | Chloral, excision of nerve, and morphine-injections. |
| 50 Kretschy ..... | Wien. Med. Woch., 1876. | M., 15 ....... | Traumatic.... | 16 | R. | ...... | Chloral and amputation of finger. |
| 51 Verneuil...... | Gaz. des Hôp., 1874, p. 94–97................ | F., adult.. ... | Traumatic ... | 5 | D. | Acute. | Chloral and morphia. |
| 52 Gatti......... | L' Independente, Sept., 1872............... | M., 40........ | Traumatic.... | 6 | R. | ...... | Chloral, bleeding and curare. |
| 53 Otto (Dorpat).. | Med. Zeitschrift, 1872, p. 188.. ............... | ............... | Cold and wet. | 13 | R. | ...... | Curare, chloral, and then curare again. |

| | | | | | | | | |
|---|---|---|---|---|---|---|---|---|
| 54 | Do. | Med. Zeitschrift, 1872, 188 | M., 24 | Traumatic | 15 | D. | ...... | Chloral, morphia, curare. |
| 55 | Sanni, Carlo | Il Raccogliatore Med., Nov. 1873 | F., 42 | Dog bite | 15 | R. | ...... | Chloral, morphia, curare. |
| 56 | Ghio, Ant. | Gaz. Lomb. xxxiv. 30,'74 | M., 16 | Traumatic | 17 | R. | ...... | Chloral, curare, warm baths. |
| 57 | Kennedy, S. | Phila. M. & S. Reporter, Feb., 1873 | M., 52 | Traumatic | 11 | D. | ...... | Chloral and calabar. |
| 58 | Bennett, W. H. | Phila. Med. Times, Dec., 1871 | M., 18 | Traumatic | 7 | D. | ...... | Chloral, calabar bean, chloroform. |
| 59 | McEwan, J. | Glasgow Jour., Oct., 1874 | M., 10 | Traumatic | 21 | D. | ...... | Chloral, calabar bean, ice, chloroform. |
| 60 | Porter | Am. Jour. Med. Science, July, 1876 | ...... | Traumatic | 13 | R. | ...... | Chloral and potass. bromid. |
| 61 | Wood, H. C. | Phila. M. & S. Reporter, Oct., 1877 | ...... | ...... | ...... | R. | ...... | Chloral and bromide in large doses. Blister to neck. |
| 62 | Blachez | Gaz. Hebdom. 1878 | M., | Cold and wet. | ..... | R. | ...... | Chloral, bromide potass. and baths. |
| | | | M., | Wet | 1 | R. | ...... | Chloral, bromide potass. and baths. |
| 63 | Evans, H. G. | Phila. Med. Times, May, 1878 | ...... | Traumatic | 11 | R. | ...... | Chloral, bromide potass. and baths. |
| 64 | Panthel | Berlin, Klin. Woch., '78 | ...... | Traumatic | 5 | R. | ...... | Chloral, bromide. Later, chloral. |
| 65 | Heath | Lancet, March 23, 1878 | M., 45 | Traumatic | 14 | R. | ...... | Chloral, bromide. Later, chloral. |
| 66 | Carruthers, J. B | Edinboro Med. Journal, March, 1877 | M., 14 | Traumatic | 13 | R. | Acute. | Chloral, bromide. Later, chloral. |
| 67 | Tuxford, Arth. | Schmidt's Jahrbücher, 171, p. 239 | ...... | Wasp stings | 3 | R. | ...... | Chloral, bromide. Later, chloral. |
| 68 | Samuels | Lancet, Feb. 16, 1878 | M., 17 | Traumatic | 7 | R. | ...... | Chloral, bromide. |
| 69 | Krause, R. | Inaug. Dissert., Breslau, 1878 | F., 21 | Traumatic | 14 | R. | ...... | Ch'l., potass. brom., morph. & curare. |
| 70 | Ferrini, Giov. | Gaz. de Paris, 1876 | M. (negro) | Traumatic | 5 | R. | ...... | Chloral and jaborandi. |
| 71 | Do. | Gaz. Lombardi, 1877 | ...... | Wet & wound. | 2 | R. | ...... | Chloral and jaborandi. |
| 72 | Bannister, H. M | Quoted by Kerecht | M., 24 | Traumatic | 13 | D. | ...... | Chloral and other remedies. |
| 73 | Dawson, B. | Am. Jour. Obstet., April, 1876 | F., | After operation | 9 | D. | ...... | Chloral subcutaneously, calabar bean. |
| 74 | Foster | Lancet, April, 1871 | M., 40 | Traumatic | 16 | R. | ...... | Chloral and amyl. |
| 75 | Dawson, W. W. | The Clinic, July, 1876 | ...... | Traumatic | 16 | R. | ...... | Chloral, cal. bean, morphine and opium |
| 76 | Calastri | Gaz. Lombardi | M., 17 | Traumatic | 19 | R. | ...... | Chloral, chloroform, opium, extension. |
| 77 | ...... | Pacific M. & S. Journal; Lancet, March 24, 1874 | M., 9 | Traumatic | ...... | R. | ...... | Chloral, tr. opii., potass. bromid. and ice to spine. |

TABLE II.—*Continued.*

| Name of Reporter. | Journal References. | Age and Sex. | Cause. | Kind. | Result. | Date after Injury. | Treatment. |
|---|---|---|---|---|---|---|---|
| 78 Drake ....... | Practitioner, 1877, p. 52.. | M., 28........ | Traumatic.... | 13 | D. | ...... | Chloral and cal. bean; nerve stretching. |
| 79 Gay, G. W.... | Boston M. & S. Journal, Aug. 1876 ........... | M., 54........ | Traumatic.... | 10 | D. | .. .. | Chloral and potass. bromide. |
| 80 Bresson....... | Practitioner, 1877, p. 142 | Man ......... | From burns... | 8 | D. | ...... | Chloral, morphia, calomel and hot baths. Died from laryngeal spasm from cold air after a hot bath. |
| 81 Verneuil...... | Gaz. des Hôp., No. 43, '70 | Man . ....... | Traumatic.... | 8 | R. | ..... | Chloral and morphia. |
| 82 Do......... | Do do | M., 20........ | Traumatic.... | 15 | D. | ... .. | Chloral, morphia, potass. bromid. |
| 83 Haynes, W. H. | (Letter to the Author)... | Infant, 9 days. | T. Nascentium. | ...... | D. | ...... | Chloral and potass. bromid. |
| 84 Parrish, Jos... | Do do .. | M., 9......... | Traumatic.... | 10 | R. | ...... | Chloral, potass. bromid., morphia; then after two weeks, morphia alone. |
| 85 Johnson...... | Med. Times & Gazette, June, 1875........... | M., 28........ | Traumatic.... | 8 | D. | Acute. | Chloral and calabar bean. |
| 86 Imray ....... | Med. Times & Gaz., May 27, 1876............. | M. (negro), 47. | Traumatic.... | 13 | R. | ...... | Chloral and opium. |
| 87 Boon, A. P.... | Lancet, Feb. 16, 1878.... | M., 10........ | Traumatic.... | 36 hrs. | D. | ...... | Chloral and cannabis indica. |
| 88 Do....... | Do do .... | M., 18........ | Traumatic.... | 48 hrs. | R. | ...... | Chloral and cannabis indica. |
| 89 Do....... | Do do .... | M., 50........ | Traumatic.... | 6 | R. | ...... | Chloral and cannabis indica. |
| 90 Do...... | Do do .... | M., 10........ | Idiopathic.... | ...... | R. | ...... | Chloral and cannabis indica. |
| 91 Do....... | Do do ... | M., 14........ | Idiopathic.... | ...... | R. | ... .. | Chloral and cannabis indica. |
| 92 Davis, F. H... | Do March 23, 1878.. | F., 10 ........ | Idiopathic.... | 10 | D. | ...... | Chloral, chloroform, morphia. Chloral seemed to give most relief. |
| 93 Barruch, S.... | (Letter to the Author)... | M. (negro) ad'lt. | .............. | ...... | R. | ...... | Chloral and potass. bromid. |
| 94 Mr. Cock. ... | Taylor, Guy's Hosp. Reports, 1878, p. 339..... | M., 23........ | Traumatic.... | 7 | D. | Acute. | Chloral and morphia. |
| 95 Dr. Fagge.... | Taylor, Guy's Hosp. Reports, 1878, p. 339.... | M., 20........ | Traumatic.... | 5 | R. | Chr. | Chloral and potass. iodid. |
| 96 Drs. Davies & Colley...... | Taylor, Guy's Hosp. Reports, 1878, p. 339.... | M., 13........ | Traumatic.... | 5 | D. | Acute. | Chloral, opium and potass. bromide. |

Total cases, 96 { Recovered......63

Died.............33 { Males.........23; Females .... 7; Infants ..... 1 }

Died............ {

Traumatic..........................28

Idiopatic ............................ 3

Tris. Nasc.......................... 1

Puerperal ........................... 1

—

Total..........................33 }

MIXED TREATMENT.

| REMEDIES USED. | RECOVERIES. | DEATHS. | TOTAL. |
|---|---|---|---|
| Chloral and potass. bromid................ | 16 | 5 | 21 |
| Chloral, potass. bromide and calabar bean.. | 1 | 1 | 2 |
| Chloral, potass. bromid. and morphine...... | 4* | 4 | 8 |
| Chloral, potass. bromid., morph. and woorara | 1 | 0 | 1 |
| Chloral, potass. bromid, morph. and atropia. | 1* | 0 | 1 |
| Chloral, potass. bromid. and chloroform.... | 2 | 0 | 2 |
| Chloral and physostigma.................... | 4 | 5 | 9 |
| Chloral, physostigma and morphine........ | 1 | 0 | 1 |
| Chloral, physostigma and chloroform..... | 1 | 2 | 3 |
| Chloral, curare and morphine............. | 1 | 1 | 2 |
| Chloral and curare ....................... | 2 | 0 | 2 |
| Chloral, curare and bleeding............ | 1 | 0 | 1 |
| Chloral, chloroform and morphine..... ... | 1 | 3 | 4 |
| Chloral and chloroform.................... | 0 | 2* | 2 |
| Chloral and atropia......................... | 3 | 1 | 4 |
| Chloral, atropia and morphine............ | 0 | 1 | 1 |
| Chloral and morphine........................ | 9* | 5 | 14 |
| Chloral, aconite and morphine............. | 1 | 0 | 1 |
| Chloral and amyl.............................. | 1 | 0 | 1 |
| Chloral and jaborandi....................... | 3 | 0 | 3 |
| Chloral and nicotine.......................... | 1 | 0 | 1 |
| Chloral and cannabis indica................. | 5 | 1 | 6 |

* In one case there was surgical interference. In some of these cases marked "morphine" opium was used; and in some marked "atropia," belladonna was employed.

4. The long uninterrupted sleep at night. In this particular there is a resemblance to chorea. In true tetanus there is no such relief until convalescence. It is noticeable also that no such relief followed the administration of chloral until it was given in thirty-grain doses.

Dr. D. W. Yandell (American Practitioner)* comes to the following conclusions from a study of 400 cases:

1. Tetanus occurs in males in proportion of four to one, and tends to recovery oftenest in females.

2. Most fatal in persons under 10 years of age—least fatal between 10 and 20 years.

3. Traumatic tetanus usual from four to nine days after injury, and these cases represent the largest mortality.

4. Recovery usual when disease appears after the ninth day.

5. When symptoms last fourteen days, recovery the rule, death the exception, *apparently independent of treatment.*

6. Tetanus appearing in puerperal state is most fatal.

7. Chloroform up to this time has yielded the largest percentage of cures.

With reference to the second clause of Dr. Yandell's first proposition, I think there must be a mistake, for from the statistics which I have collected, more females die in proportion to the number attacked than males:

| AGE. | FIRST TABLE. | | SECOND TABLE. | | TOTAL. | |
|---|---|---|---|---|---|---|
| | DEATH. | RECOVERY. | DEATH. | RECOVERY. | DEATH. | RECOVERY. |
| First 10 years of life.......... | 5 | 6 | 7 | 19 | 12 | 25 |
| 10 to 20 years.................... | 5 | 19 | 10 | 17 | 15 | 36 |
| 20 to 30 years.................... | 9 | 8 | 11 | 16 | 20 | 24 |
| 30 to 40 years.................... | 7 | 8 | 18 | 16 | 25 | 24 |
| 40 to 60 years.................... | 4 | 5 | 5 | 13 | 9 | 18 |
| 60 to 70 years.................... | ............ | ............ | 2 | ............ | 2 | ............ |
| Totals ........................ | 30 | 46 | 53 | 81 | 83 | 127 |

* Detroit Review of Med., May, 1875.

| DATE OF TETANUS AFTER INJURY. | FIRST TABLE. | | SECOND TABLE. | | TOTAL. | |
|---|---|---|---|---|---|---|
| | DEATH. | RECOVERY. | DEATH. | RECOVERY. | DEATH. | RECOVERY. |
| First 9 days | 16 | 18 | 28 | 33 | 44 | 51 |
| From the 9th to 21st day | 13 | 28 | 19 | 29 | 32 | 57 |
| From the 20th day on | 1 | 3 | 3 | 13 | 4 | 16 |
| Totals | 30 | 49 | 50 | 75 | 80 | 124 |

| SEX IN CASES OF DEATH. | FIRST TABLE. | | SECOND TABLE. | | TOTAL. | |
|---|---|---|---|---|---|---|
| | CASES. | DEATHS. | CASES. | DEATHS. | CASES. | DEATHS. |
| Males | 62 | 23 | 107 | 45 | 169 | 68 |
| Females | 16 | 7 | 22 | 12 | 48 | 19 |
| Totals | 78 | 30 | 129 | 57 | 207 | 87 |

The period at which tetanus seems to have been the most fatal was in patients from 20 to 30 and from 30 to 40 years of age. This differs somewhat from Dr. Yandell's conclusions, but the study of a larger number of cases may entirely change the figures.

As I said before, I believe that we have in chloral hydrate one of the most useful agents known for the treatment of this terrible disease, but I also maintain that in using it alone we are neglecting to do our full duty. A combination of chloral hydrate, potass. bromid., morphia and calabar bean would seem to be that best suited to the majority of cases. Removal of foreign bodies, maintaining the bowels in a relaxed condition, exclusion of light and maintenance of perfect quiet, with plenty of stimulus and liquid food, are not to be neglected. Quinine should be given in large doses if the temperature is high, and chloroform may be given by inhalation to modify the severity of the spasms.

Should the glottic spasm despite these measures, be so severe as to threaten life (and it often does), tracheotomy should be resorted to at once.

Whatever the drug employed in this disease, it must be given in large amounts, frequently repeated. Many of the cases in both tables, but especially the first, were undoubtedly lost owing to the fact that the drug was not given in sufficiently large doses. Ordinary "large doses" are out of the question. Those only succeed that would in other diseases be considered *enormous*. As much as 1,140 grains have been given in twenty-four hours to a child æt. 12 years with only good results.

Prof. H. C. Wood,* of Philadelphia, speaks highly of the use of bromide of potassium in this disease. He cites eighteen cases, in two of which deaths occurred. He says: "I have been unable to find a recorded death from the disease after the free exhibition of the bromide, although according to Dr. Roemer one or two have occurred." It is for this reason that I believe that this drug should be combined with the chloral. To have any effect both of the drugs must be given in *very large doses*, the tetanic spasm seeming to antagonize the effects of the drugs. Some of the cases of death recorded in these tables might, undoubtedly, have been written recoveries had the drug been used with sufficient freedom.

Chloral has been used in another way by Dr. Macnamara.† Believing that it exercises no control over the spasms, he gives it at night in 40-grain doses and an additional dose in the morning if the temperature is above 101° F. No other medicine is given. Patient is made to take four ounces of brandy with milk every four hours. He claims to have saved seventeen in twenty consecutive cases (traumatic?) occurring in natives of India.

When it is impossible to give it by the mouth, it may be given by the rectum in milk. It has been given subcutaneously with a resulting cure by Dr. J. Hyde Salter‡ and others§. The rectum, however, affords every advantage when it is impossible to give it by the mouth. Intravenous injections of chloral which have been tried in France‖ are not justifiable, unless an immediate and decided effect is necessary to save life.

---

* Materia Medica and Therapeutics, Phila., 1877, p. 313.

† (Practitioner, Nov., 1874). Quoted by H. C. Wood, op. cit., p. 323.

‡ Practitioner, Dec., 1876.

§ Ribell. Ann. de Gynécologie, June, 1875.

‖ Oré., Gaz. de Paris, 21, 1874. Cruveilher, Gaz. des Hôp., 49, 1874. Labbé, Gaz. de Paris, 21, 1874. Tillaux, L'Union, 101, 1874. Lannelongue, Gaz. des Hôpitaux, 125, 1874.

Dr. J. K. Bigelow* in one case of tetanus found great relief to spasm by opening the wound and putting in chloral.

Dr. Allison Maxwell,† of Indianapolis, Ind., tried this in one case, but the smart of the application was so great that the patient could not bear it, and afterwards would not allow even a weak solution of chloral to be used locally.

Mr. R. W. Findlay,‡ V. S., in two cases of traumatic tetanus in the horse, secured a favorable result by giving ʒiv potass. bromid. and ʒii chloral hydrate in ℥viii water three times a day.

191 WEST TENTH ST., New York City.

* American Practitioner, Dec., 1877.

† American Practitioner, Jan., 1880.

‡ N. Y. Med. Record, 1876, p. 613.

